# Prediction of State of Health of Lithium-Ion Battery Using Health Index Informed Attention Model

**DOI:** 10.3390/s23052587

**Published:** 2023-02-26

**Authors:** Yupeng Wei

**Affiliations:** Department of Industrial and Systems Engineering, San Jose State University, San Jose, CA 95192, USA; yupeng.wei@sjsu.edu

**Keywords:** battery, prognostics, health index, attention model

## Abstract

State-of-health (SOH) is a measure of a battery’s capacity in comparison to its rated capacity. Despite numerous data-driven algorithms being developed to estimate battery SOH, they are often ineffective in handling time series data, as they are unable to utilize the most significant portion of a time series while predicting SOH. Furthermore, current data-driven algorithms are often unable to learn a health index, which is a measurement of the battery’s health condition, to capture capacity degradation and regeneration. To address these issues, we first present an optimization model to obtain a health index of a battery, which accurately captures the battery’s degradation trajectory and improves SOH prediction accuracy. Additionally, we introduce an attention-based deep learning algorithm, where an attention matrix, referring to the significance level of a time series, is developed to enable the predictive model to use the most significant portion of a time series for SOH prediction. Our numerical results demonstrate that the presented algorithm provides an effective health index and can precisely predict the SOH of a battery.

## 1. Introduction

Lithium-ion batteries are extensively adopted as a power resource for unmanned aerial vehicles, electric mobility, and electric vehicles as a result of their low maintenance frequency, long life cycle, and high energy efficiency [1,2]. Research efforts in the field of lithium-ion batteries have focused on various aspects, including improving charge speed and energy density, reducing production costs, and extending battery life cycles [3,4]. In addition, there have been studies on the evolution of li-rich layered cathode materials [5], with a particular focus on controlling the anionic redox of li-rich layered oxides to maximize energy density [6]. Nevertheless, the safety and efficiency of batteries can reduce with the increment of time and charge-discharge cycles, which is also known as battery aging [7,8]. For example, the traveling mileage of unmanned aerial vehicles and electric vehicles can be dramatically reduced due to the battery aging issue. The battery aging issue in electric mobility may result in severe fires and outbursts. As a result, it is necessary to monitor the health condition of batteries so that a proper maintenance schedule can be made to reduce the negative impact of battery aging.

State of health (SOH) represents the primary measurement that provides reliable information to monitor and predict the health conditions of batteries [9]. SOH is defined as the rate between the peak battery capacity and its rated capacity [10]. In the literature, numerous algorithms have been developed to make SOH predictions, and these algorithms can be categorized as model-based and data-driven algorithms. The model-based algorithms are built upon electronic, chemical, and mathematical models [11]. The primary drawback of the model-based algorithms is that the prediction performance can not be guaranteed as these methods can not capture the complex relationships among the enormous electrical and chemical components of batteries. To address the limitation of the model-based algorithms, data-driven algorithms are increasingly used in SOH predictions and this work will aim attention at data-driven algorithms. Data-driven algorithms are built upon statistical or machine-learning algorithms, such as Kalman filter [12], Gaussian Process [13], and deep learning methods [14]. While promising, most of the existing data-driven algorithms are not effective in dealing with time series as they are not able to utilize the most significant part of a time series while predicting the SOH of a battery. To address this issue, an attention-based deep learning predictive algorithm is developed in this work, where an attention matrix referring to the significance level of a time series at distinctive time is generated so that the deep learning predictive model can utilize the most relevant portion of a time series for SOH predictions. Moreover, it has been demonstrated that constructing an effective health index can increase the performance of a predictive model [15,16]. However, to our best knowledge, very few studies have been conducted to define or construct a health index of a battery. Although a few studies have been reported on the development of a health index for complex systems and machinery equipment [17], such as aircraft engines and bearings, these methods can only construct a monotonically decreasing health index which is not capable of capturing capacity fade and capacity regeneration behaviors of a battery. To fill this gap, we propose four properties to capture capacity degradation and regeneration behaviors. We also develop a convex optimization model to obtain a health index of a battery. The major contributions of this work can be described as below:

- A convex optimization model is introduced to obtain a health index of a battery, such an index can accurately capture the degradation trajectory of a battery as well as improves the SOH prediction performance.
- An attention-based deep learning predictive algorithm is presented, where an attention matrix referring to the significance level of a time series is adopted in SOH predictions so that the predictive algorithm can utilize the most significant portion of a time series for SOH predictions.

The remaining sections of this work is arranged as follows. Section 2 reviews the data-driven algorithms in SOH predictions. Section 3 presents the proposed convex optimization model for learning a health index and an attention-based deep learning predictive algorithm for SOH predictions of batteries. Section 4 demonstrates the efficiency of the proposed algorithm. Section 5 draws a conclusion and provides a discussion of future work.

## 2. Data-Driven Algorithms for SOH Predictions

The data-driven algorithms used for predicting state of health (SOH) of batteries include filter-related models and machine-learning models. The filter-related models incorporate techniques such as the Kalman filter [18,19], particle filter [20,21], and others. For instance, Chen et al. [22] developed an unscented Kalman filter to evaluate the SOH of a battery. Their presented Kalman filter incorporated the internal resistance rate to trace the subsequent SOH trajectory while making predictions. The results showed that the presented Kalman filter could estimate the SOH with a low prediction error rate. Dong et al. [23] utilized the particle filter for estimating the SOH of a battery. Their proposed filter was integrated with a stochastic model to track abrupt changes in the process of battery capacity fade. The results demonstrated that the particle filter was able to predict the SOH of multiple battery cells with relatively high accuracy. The primary advantage of the filter-based methods is their self-correlation capability. However, these methods have difficulty in considering multiple battery cells while making SOH predictions [24].

To better utilize all historical information from all battery cells, machine learning algorithms, especially deep learning algorithms are increasingly adopted. These algorithms incorporate random forests [25], long short-term memory (LSTM) [26], temporal convolutional network (TCN) [27], graph convolutional network (GCN) [28,29], and so on. For example, Mawonou et al. [30] presented a novel random forests SOH estimation algorithm to consider both the usage pattern of drivers and the environmental conditions of electric vehicles. In order to boost the estimation performance, two aging indicators were also incorporated into SOH estimation. The numerical results have manifested that the present novel random forests algorithm can reach a prediction error of 1.27%. Zhao et al. [31] combined the LSTM algorithm with the Gaussian process to evaluate the SOH of a battery, where several health factors extracted from sensor data obtained from charge cycles were utilized. The LSTM model was adopted to track the trajectory change of extracted health factors and the trajectory change was fed into the Gaussian process model for SOH predictions. A publicly available battery dataset was utilized to evaluate the efficacy of the presented algorithm. Zhou et al. [32] used the TCN for battery SOH predictions, where the dilated convolutional operation and causal operation were primarily used in the TCN model. In addition to TCN, the empirical mode decomposition was adopted to reduce the nuisance factors so that the SOH prediction performance can be more robust. Wei and Wu [33] presented a GCN algorithm to predict the SOH and the remaining useful charge and discharge cycles of batteries, where a two-phase optimization model was presented to develop a graph with optimized graph entropy and density. Numerical results have illustrated that the presented GCN algorithm could predict the SOH accurately even without using sensor data during the charging process.

In summary, numerous data-driven algorithms have been developed for predicting the SOH of a battery. However, most of these algorithms cannot effectively learn a health index that captures the degradation trajectory of a battery. Additionally, current data-driven algorithms are not able to handle time series data as they do not use the most significant portion of the time series when predicting the SOH of a battery. To address these issues, we first developed a convex optimization model to obtain a health index of a battery. This health index accurately captures the degradation trajectory of a battery and improves the prediction performance of SOH. We also introduce an attention-based deep learning predictive model, where an attention matrix is generated to show the significance level of each time step in the time series. This allows the deep learning predictive model to utilize the most relevant portion of the time series data when predicting SOH.

## 3. Health Index Informed Attention Model

The proposed algorithm comprises of three primary steps. Firstly, temporal features are extracted from the sensor data collected during the charging and discharging cycles of lithium-ion batteries. Secondly, a convex optimization model is constructed, and the extracted temporal features are given to the presented optimization model to learn health indices of batteries. Lastly, the extracted features and the learned health index are used to train an attention-based deep learning model for predicting SOH. The following sections provide detailed descriptions of these three steps.

### 3.1. Temporal Features Extraction

**Figure 1:**
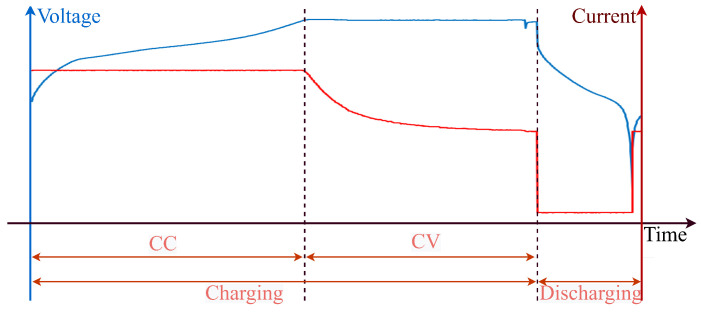
The current and voltage measurements in a single charge and discharge cycle.

The most common battery health monitoring data includes current, voltage, and temperature. Figure 1 shows the current and voltage measurements during a single charge and discharge cycle. In a charge cycle, the battery is subjected to a consistent current (CC) until the measured voltage reaches a specific value, and then under a consistent voltage (CV) until the current drops to a specific value. In the discharge cycle, the battery is loaded under a consistent current condition until the battery voltage drops to a certain range. The recurrent charging and discharging process leads to a reduced capacity of lithium-ion batteries, which can be observed through changes in sensor measurements. For instance, as batteries age, they require more time to reach a specific voltage during the charge cycle, compared to new batteries. In this work, temporal features are derived from current, voltage, and temperature measurements to capture the depreciation behaviors in charge and discharge cycles.

In the charge cycle, one temporal feature extracted from voltage measurement is written as Equation (1),

(1) $$\min\;t\left(s\right)\;s.t.\;V_{t(s)}-V_{max}\geq 0$$

where t(s) refers to the time in the *s*-th sampling period; $V_{t(s)}$ refers to the voltage measurement at time t(s); and $V_{max}$ is the maximum voltage of a battery. This extracted feature refers to the time to the maximum voltage of batteries. The second feature extracted from voltage measurement is written as Equation (2),

(2) $$\max\;t\left(s\right)-t\left(s^{\prime }\right)\;s.t.\;V_{t(s)}-V_{max}\geq 0,V_{t(s^{\prime })}-V_{max}\geq 0$$

where t(s) and $t(s^{\prime })$ represent the time in the *s*-th and $s^{\prime }$-th sampling period; and $V_{t(s)}$ and $V_{t(s^{\prime })}$ are the measured voltage at t(s) and $t(s^{\prime })$. This feature refers to the time that a battery stays in CV mode. The first feature extracted from the current measurement can be written as Equation (3),

(3) $$\max\;t\left(s\right)-t\left(s^{\prime }\right)\;s.t.\;A_{t(s)}=A_{t(s^{\prime })}=A_{constant}$$

where $A_{t(s)}$ and $A_{t(s^{\prime })}$ are the current measurement at t(s) and $t(s^{\prime })$, and $A_{constant}$ represents the current that the battery is under the CC condition. This extracted feature refers to the time that a battery is carried out under the CC condition. The second feature extracted from the current measurement is written as Equation (4),

(4) $$\min\;t\left(s\right)-\max\;t\left(s^{\prime }\right)\;s.t.\;A_{t(s)}\leq A_{constant},A_{t(s^{\prime })}\leq A_{min}$$

where $A_{min}$ is the minimum current of the battery. This feature represents the time to the minimum current of batteries. The feature extracted from temperature measurement can be written as Equation (5),

(5) $$\min\;t\left(s\right)\;s.t.\;T_{t(s)}=\max\left\{T_{t(s)},\forall s\right\}$$

where $T_{t(s)}$ is the temperature at time t(s). This feature represents the time to the maximum temperature in the charge cycle.

In the discharge cycle, the feature extracted from voltage measurement can be represented in Equation (6),

(6) $$\min\;t\left(s\right)\;s.t.\;V_{t(s)}\leq V_{min}$$

where $V_{min}$ represents the minimum voltage of a battery in the discharge cycle. This extracted feature refers to the time to the minimum voltage. The feature extracted current measurement in the discharge cycle is written as Equation (7),

(7) $$\min\;t\left(s\right)\;s.t.\;A_{t(s)}\geq A_{load}$$

where $A_{load}$ is the battery load while discharging. This feature represents the discharging time. The last feature extracted from temperature measurement in the discharge cycle can be written as Equation (8), and this feature refers to the time to the maximum temperature in the discharge cycle.

(8) $$\min\;t\left(s\right)\;s.t.\;T_{t(s)}=\max\left\{T_{t(s)},\forall s\right\}$$

These extracted features are fed into the proposed convex optimization model to obtain the health index of a battery.

### 3.2. Health Index Generation of a Battery

In the literature, few studies have been conducted on learning monotonically decreasing health indices for complex engineering systems, such as those described in the Ref. [34]. While these health indices have proven effective in solving prognostic problems, they are unsuitable for modeling the degradation behavior of lithium-ion batteries. For instance, the capacity of a battery does not decrease monotonically due to phenomena such as capacity regeneration. To better characterize the degradation behavior of lithium-ion batteries, we introduce four attributes for learning health indices. The first two attributes are based on the physical properties of aging batteries, while the remaining attributes are commonly used to capture the general degradation behaviors of complex systems [35].

- Attribute 1: The health indices of aging batteries should be piece-wise monotonically decreasing with the increasing number of charge and discharge cycles.
- Attribute 2: The health indices of aging batteries should increase after a complete charge-discharge cycle and a long period of storage.
- Attribute 3: The variance of the failure threshold for the health indices of aging batteries should be minimal.
- Attribute 4: The health indices should be consistent with the true capacity degradation trajectory of batteries.

The continuously repeated charge and discharge cycles lead to the reduced health conditions of lithium-ion batteries; therefore, Attribute 1 aims at learning piece-wise monotonically decreasing health indices in the piece-wise continued charging and discharging process. A battery may regain some capacity because of the capacity regeneration phenomenon; therefore, Attribute 2 aims at learning increasing health indices after a complete charge-discharge cycle and a long period of storage. Moreover, Attribute 3 and Attribute 4 are introduced to learn consistent and reliable health indices with less variance. Based on these four attributes, an optimization model is introduced as Equation (9),

(9) $$\begin{array}{cc} \min & \;\theta_{1}\sum_{i=1}^{m}\sum_{j=1}^{n_{i}}\sum_{k=1}^{l}w_{k}\left(f_{i,j,k}-f_{i,j-1,k}\right)p_{i,j}+\;\theta_{2}\sum_{i=1}^{m}\sum_{j=1}^{n_{i}}\sum_{k=1}^{l}w_{k}\left(f_{i,j-1,k}-f_{i,j,k}\right)r_{i,j}+\\ \; & \;\theta_{3}w^{T}Q^{T}MQw+\theta_{4}{\left(\sum_{i=1}^{m}\sum_{j=1}^{n_{i}}\sum_{k=1}^{l}{\left(w_{k}f_{i,j,k}-y_{i,j}\right)}^{2}\right)}^{1/2}\\ s.t. & \;\sum_{a}\theta_{a}=1;\;i=1,\ldots ,m,\;j=1,\ldots ,n_{i},\;k=1,\ldots ,l \end{array}$$

where *m* refers to the total number of battery units; $n_{i}$ refers to the total number of cycles for the battery cell *i*; *l* is the total number of temporal features are extracted; $w_{k}$ is the weighted coefficient for feature *k* to combine extracted features to learn health indices of batteries; $f_{i,j,k}$ represents the *k*th feature derived from the battery cell *i* in cycle *j*; $f_{i,j-1,k}$ represents the *k*th feature derived from the battery cell *i* in cycle j−1; $p_{i,j}$ and $r_{i,j}$ are binary variables represent the information of storage periods; $p_{i,j}=1$ and $r_{i,j}=0$, if the cycle *j* is not after a storage period; $p_{i,j}=0$ and $r_{i,j}=1$, if the cycle *j* is after a storage period; $y_{i,j}$ is the battery capacity for the battery cell *i* in cycle *j*; $w\in R^{l\times 1}$ is the vector stores the weighted coefficients $w_{k}$; $Q\in R^{m\times l}$ is the matrix recording the temporal features extracted from failure observations with rows referring to each battery cell and the columns referring to every extracted features *k*; $\theta_{a},\forall a$ are hyperparameters that decide the importance of four proposed attributes; M=(I−O/j)/(j−1), $O\in R^{m\times m}$ is a matrix with all elements are one, and $I\in R^{m\times m}$ is an identity matrix; the matrix M is introduced to estimate the unbiased variance of the failure threshold of health indices, which is proved as Equation (10). Similar proofs can be found in the Refs. [36,37].

(10) $$\begin{array}{cc} \; & \left({\left(Qw\right)}^{T}Qw-m{\left(\left(1^{T}Qw\right)/m\right)}^{2}\right)\\ \; & =w^{T}Q^{T}\left(\left(I-11^{T}/m\right)/\left(m-1\right)\right)Qw/\left(m-1\right)=w^{T}Q^{T}MQw \end{array}$$

To simplify the proposed optimization model, this model can be rewritten as Equation (11),

(11) $$\min\;\theta_{1}p^{T}Dw-\theta_{2}r^{T}Dw+\theta_{3}w^{T}Q^{T}MQw+\theta_{4}{∥Fw-y∥}_{2}^{2}\;s.t.\;\sum_{a}\theta_{a}=1$$

where $p\in R^{1\times \sum_{i}n_{i}-1}$ is a vector stores $p_{i,j}$ for all i,j and $p=[p_{1,1},\ldots ,p_{1,n_{1}},p_{2,1},\ldots ,p_{2,n_{2}},\ldots ,$$p_{m,n_{m}}{]}^{T}$; $r\in R^{1\times \sum_{i}n_{i}-1}$ is a vector stores $r_{i,j}$ for all i,j and $r=[r_{1,1},\ldots ,r_{1,n_{1}},r_{2,1},\ldots ,r_{2,n_{2}},\ldots ,$$r_{m,n_{m}}{]}^{T}$; $D\in R^{\sum_{i}n_{i}-1\times m}$ is a matrix of collecting the differences of adjacent sampling time, and $D={\left[D_{1},\ldots ,D_{i},\ldots ,D_{m}\right]}^{T}$; $F\in R^{\sum_{i}n_{i}\times m}$ is a matrix recording the extracted features for all battery units and cycles, and $F={\left[F_{1},\ldots ,F_{i},\ldots ,F_{m}\right]}^{T}$. The matrix $D_{i}$ and the matrix $F_{i}$ can be written as Equation (12);

(12) $$D_{i}=\left(\begin{array}{ccc} f_{i,2,1}-f_{i,1,1} & \cdots & f_{i,2,l}-f_{i,1,l}\\ f_{i,3,1}-f_{i,2,1} & \cdots & f_{i,3,l}-f_{i,2,l}\\ \vdots & \ddots & \vdots \\ f_{i,n_{i},1}-f_{i,n_{i}-1,1} & \cdots & f_{i,n_{i},l}-f_{i,n_{i}-1,l} \end{array}\right)\;F_{i}=\left(\begin{array}{cccc} f_{i,1,1} & f_{i,1,2} & \cdots & f_{i,1,l}\\ f_{i,2,1} & f_{i,2,2} & \cdots & f_{i,2,l}\\ \vdots & \vdots & \ddots & \vdots \\ f_{i,n_{i},1} & f_{i,n_{i},2} & \cdots & f_{i,n_{i},l} \end{array}\right)$$

Moreover, $y\in R^{\sum_{i}n_{i}\times 1}$ is a vector recording the battery capacity for all battery units and cycles, where $y={\left[y_{1},y_{2},\ldots ,y_{m}\right]}^{T}$ and $y_{i}={\left[y_{i,1},y_{i,2},\ldots ,y_{i,l}\right]}^{T}$. Next, the gradient descent method could be adopted to resolve the optimization model, the optimized weighted coefficients $w_{k},\forall k$ are used to combine the extracted features to obtain the health indices. The health indices for unit *i* in the cycle *j* can be mathematically represented as $h_{ij}=\sum_{k}w_{k}f_{ijk}$.

**Figure 2:**
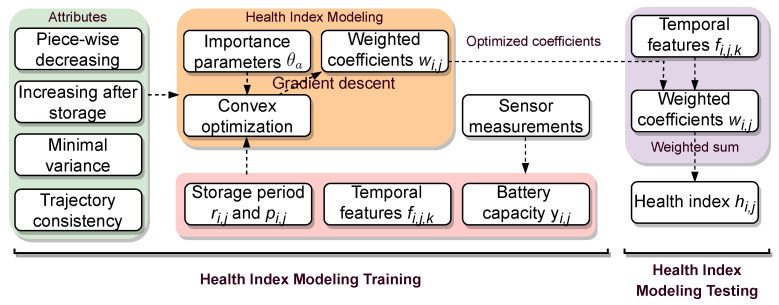
The framework of the proposed optimization model for generating the health index of a lithium-ion battery.

Figure 2 exhibits the framework of the presented optimization model for generating the health index of a lithium-ion battery. During the training process, four attributes are first introduced to capture the capacity degradation and regeneration phenomenon of a battery, and a convex optimization model is constructed based on these attributes. To train the optimization model, the importance hyperparameters $\theta_{a},\forall a$, the storage period inputs $r_{i,j},p_{i,j},\forall i,j$, temporal features $f_{i,j,k},\forall i,j,k$, and the battery capacity $y_{i,j},\forall i,j$ are given to the presented convex optimization model. The gradient descent method is adopted to learn the weighted coefficients $\omega_{i,j},\forall i,j$ in the optimization model. During the process of learning the health index, the learned weighted coefficients are used to combine the extracted temporal features to learn the health index $h_{i,j},\forall i,j$. Next, both the learn health index and the temporal features are fed into the proposed attention-based deep learning model for SOH predictions of batteries.

### 3.3. Attention-Based Deep Learning Model

While attention-based models have been used to deal with time series, most of these studies focus on neural machine translation and text generation [38,39]. The general concept of the attention model is to simulate the attention mechanism of humans used for tasks such as reading and visualization. The attention model can be represented as the value retrieval procedure of a data administration system. In such a system, each value $v_{i}$ has a key $k_{i}$ associated with it. For a query *q*, the attention value $\alpha_{i}$ on item *i* can be written as $\alpha_{i}=exp\left(sc_{i}\right)/\sum_{i}sc_{i}$, where $sc_{i}$ refers to the alignment score between the query *q* and the key $k_{i}$ for item *i*. The most often used alignment score function include dot-product [40], scaled dot-product [41], and additive [38].

**Figure 3:**
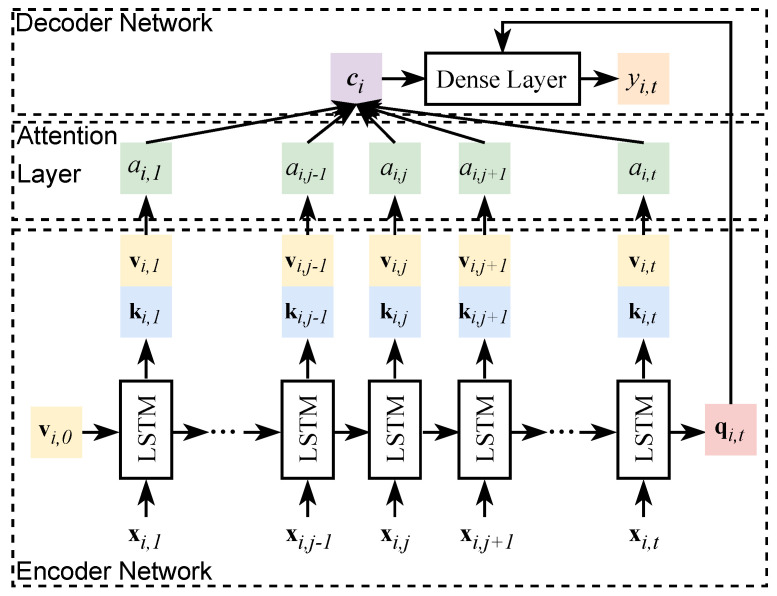
The attention-based sequence-to-one LSTM predictive model. $x_{i,j}$ refers to the vector of inputs for battery cell *i* in cycle *j*; $v_{i,j}$ is the key and $k_{i,j}$ is the value generated from the LSTM network for cell *i* in cycle *j*; $\alpha_{i,j}$ refers to the attention value for cell *i* in cycle *j*; and $c_{i}$ represents the context vector.

In this work, the attention model is integrated with a sequence-to-one LSTM neural network to predict the SOH. Figure 3 shows the attention-based sequence-to-one LSTM predictive model for SOH predictions. This predictive model consists of three primary components, including an encoder network, an attention layer, and a decoder network. In the encoder network, time series inputs $x_{i,j}$ are given to the LSTM to derive the corresponding key $k_{i,j}$ and value $v_{i,j}$. More specifically, $x_{i,j}$ is the vector that stores the extracted features and the learned health indices for battery unit *i* in cycle *j*; $v_{i,0}$ is the randomized initial input of the encoder network; $v_{i,j}$ is the value generated from the LSTM network for cell *i* in cycle *j*; $k_{i,j}$ refers to the key generated from the LSTM network for unit *i* in cycle *j*; both the $v_{i,j}$ and $k_{i,j}$ are the hidden state of the LSTM in cycle *j*, and we set $v_{i,j}$ = $k_{i,j}$ to represent that the value is identical with the key; $q_{i,t}$ refers to the query and $q_{i,t}=k_{i,t}=v_{i,t}$. In the attention layer, we use the local attention model with the monotonic alignment. The attention value $\alpha_{i,j}$ for cycle *j* can be calculated by Equation (13), where $sci_{i,a}$ refers to the alignment score between the query $q_{i,t}$ and the key $k_{i,t}$ Moreover, *d* represents the window length of the local attention model.

(13) $$\alpha_{i,j}=exp\left(sc_{i,j}\right)/\sum_{a=j-d}^{j+d}sc_{i,a}$$

The general alignment function is utilized in this work, and the corresponding score $sc_{i,j}$ can be calculated by using Equation (14). $W_{a}\in R^{z\times z}$ is the trainable weighted matrix to calculate the alignment score, where *z* is the total number of nodes in LSTM.

(14) $$sc_{i,j}=q_{i,j}^{T}W_{a}k_{i,j}$$

In the decoder network, the context vector $c_{i}$ is calculated by using Equation (15). Then, the context vector and the output of the LSTM network are given to a fully connected layer to predict the SOH of the battery cell *i* in cycle *t*, that is, $y_{i,t}$

(15) $$c_{i}=\sum_{j=1}^{t}\alpha_{i,j}v_{i,j}$$

In comparison with the traditional LSTM predictive model, the attention-based predictive model has access to the entire time series while predicting by introducing the context vector $c_{i}$. Because the context vector represents the weighted sum of the hidden outputs generated by the time series, respectively.

## 4. Case Study

### 4.1. Dataset Description

In this work, we used four battery cells (Battery No. 5, No. 6, No. 7, and No. 18) to evaluate the efficiency of the proposed algorithm. The condition monitoring data of these four battery cells were collected by the NASA Ames Prognostics Center of Excellence (PCoE) [42], where the current, voltage, and temperature data were collected during the charging and discharging process. In the charging process, the CC mode with a current of 1.5 A was discontinued when the voltage measurement was above 4.2 V and proceeded under a CV condition unless the measured current was below 20 mA. In the discharging process, the CC mode was adopted with a current of 2A unless the measured voltage was below 2.7 V, 2.5 V, 2.2 V, and 2.5 V for Battery No. 5, Battery No. 6, Battery No. 7, and Battery No. 18, respectively. The run-to-failure experiment was conducted on these four battery cells, and the test was stopped when the capacity dropped to 70%. For all four battery cells, the maximum capacity was 2 Ah, and the test was stopped when the capacity dropped to 1.4 Ah.

**Figure 4:**
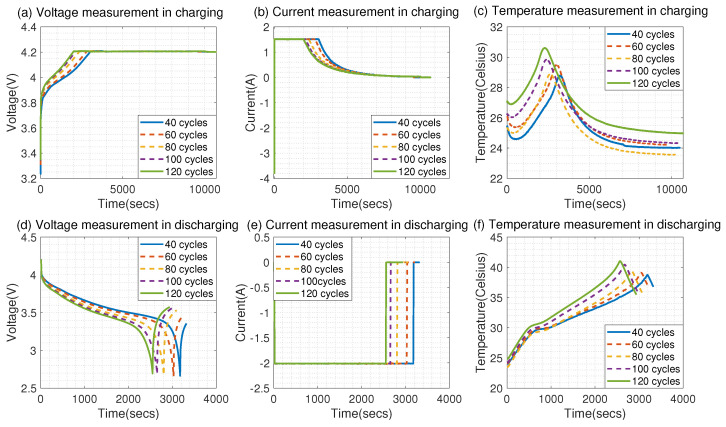
The voltage, current, and temperature measurements change over time in different charge and discharge cycles with respect to battery No. 5.

**Figure 5:**
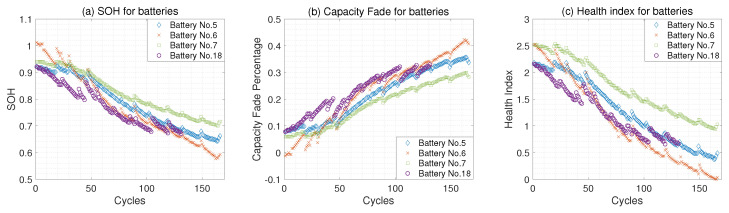
The SOH, the capacity fade, and the learned health index for batteries No. 5, No. 6, No. 7, and No. 18.

Figure 4 shows the measured voltage, current, and temperature over time during different charge and discharge cycles of battery cell No. 5. Based on this figure, we can observe that the trajectories of the measured voltage, current, and temperature change with the number of charge and discharge cycles. For example, Figure 5a shows that the time to reach the maximum voltage in 40 cycles is longer than the time to reach the maximum voltage in 120 cycles.

### 4.2. Health Index

To capture changes in battery trajectory over time, temporal features were extracted to learn the health indices of the batteries. As mentioned earlier, this paper extracted five temporal features during charge cycles and three features during discharge cycles, as they have been shown to be efficient for predicting the SOH of a battery [43]. During the charge cycles, we extracted the time to maximum voltage and temperature, time to minimum current, and time charged under CC and CV modes. During discharge cycles, we extracted the time to minimum voltage and maximum temperature, as well as time discharged under a CC mode. The extracted temporal features were then input into the optimization model presented to learn the health indices. To learn the health index of a battery, the values of hyperparameters $\theta_{a}$ for all *a* must be determined to evaluate the importance of the four proposed attributes. In this study, we considered each proposed attribute equally important by setting $\theta_{a}=0.25$ for all *a*. Furthermore, all extracted temporal features were standardized to reduce the loss of generality.

Figure 5 presents the SOH, the capacity fade, and the learned health index for batteries No. 5, No. 6, No. 7, and No. 18. Based on Figure 5, we can conclude that the learned health index shows an obvious depreciation trend, which is consistent with the degradation trajectory of the SOH. Moreover, the learned health indices can also reflect the capacity regeneration phenomenon after a long storage period. For example, the SOH of battery No. 5 increases after 150 cycles due to its storage period between the 149th and 150th charge and discharge cycles. Therefore, we can conclude that the learned health indices are consistent with the degradation trajectory of the SOH and could potentially be used for predicting the SOH.

### 4.3. SOH Estimation

**Figure 6:**
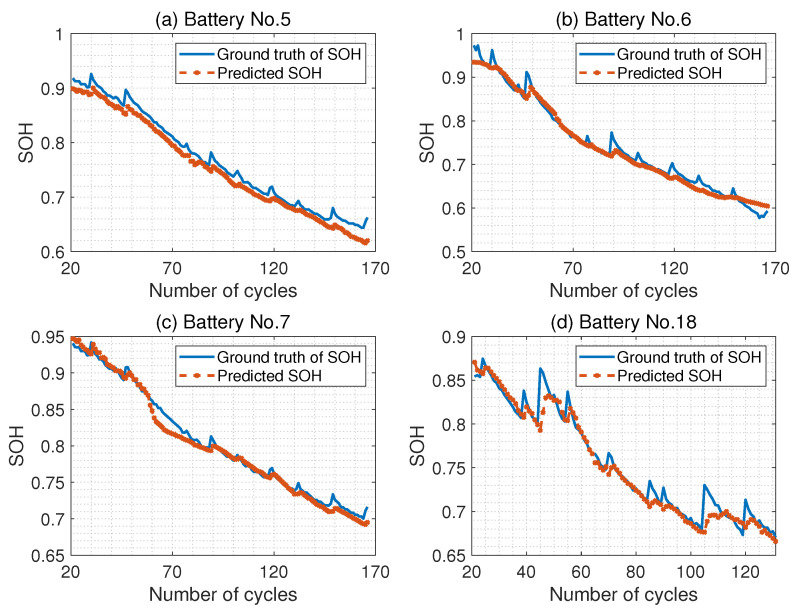
The ground truth of SOH and the predicted value for all four batteries with the prediction starting point is 20 cycles.

The proposed attention-based deep learning algorithm was then used to predict the SOH using the temporal features and learned health index. To simplify the algorithm and reduce prediction errors, the network architecture and hyperparameters used in this case study have been tabulated in Table 1. The batch size is 30, the number of hidden nodes in the LSTM layer is 50, the window size of the local attention mechanism is 5, and the learning rate is set to $1\times 10^{-5}$. Moreover, the predictive alignment is used in this case study. Figure 6 shows the ground truth of SOH and the predicted values for all four batteries, with the predicted starting point at 20 charge and discharge cycles. From Figure 6, we can see that the proposed algorithm can predict the SOH of lithium-ion batteries with satisfactory precision, as the predicted values are close to the ground truth for all four batteries. Moreover, we can also observe that the predicted SOH can capture the capacity regeneration phenomenon after a storage period.

To fully evaluate the efficacy of the proposed algorithm, it was compared with other algorithms listed in Table 2. In this table, HI-ALSTM refers to the proposed algorithm, which is the health index-informed attention-based LSTM model. HI-LSTM refers to the proposed algorithm without the attention mechanism, ALSTM is the attention-based LSTM predictive algorithm without using the health index, and LSTM refers to the classical long short-term memory without using the proposed health index or the attention mechanism. To ensure a fair comparison, the hyperparameters used in HI-LSTM, ALSTM, and LSTM are the same as those in the proposed algorithm, except that HI-LSTM does not use the attention mechanism, ALSMT does not use the learned health index, and LSTM does not use either the attention mechanism or the learned health index.

Table 3 and Table 4 show the mean absolute error (MAE) and mean absolute percentage error (MAPE) of the proposed algorithm and algorithms listed in Table 2 for all four batteries, respectively. Based on these two tables, it is evident that the learned health index and the attention mechanism can improve the accuracy of SOH prediction. For example, the average MAE of the proposed algorithm is 0.0103. However, the MAE of HI-LSTM and ALSTM is 0.0405 and 0.0121, respectively, indicating that the presented algorithm outperforms these models in terms of prediction accuracy.

Table 5 shows the root mean squared error (RMSE) of the proposed algorithm, algorithms tabulated in Table 2, and other algorithms reported in the literature [44,45] for all four batteries, to thoroughly evaluate the efficacy of the presented algorithm. The methods described in the literature incorporate logistic regression (LR) and gradient boosting decision tree (GBDT). Based on Table 5, it is evident that the learned health index and the attention mechanism can increase the SOH prediction accuracy. For example, for Battery No. 6, the prediction RMSE of the presented algorithm is 0.0153, whereas the methods without using the learned health index or the attention mechanism range from 0.220 to 0.612. With respect to Battery No. 7, the prediction RMSE of the presented algorithm is 0.0089, whereas the prediction RMSE of HI-LSTM and ALSTM is 0.0542 and 0.0145, respectively. Moreover, the presented algorithm also outperforms other methods used in the literature. For instance, the average prediction RMSE of the presented algorithm is 0.0136, whereas the average prediction RMSE of the other algorithms tabulated in Table 5 ranges from 0.0150 to 0.0457.

## 5. Conclusions and Future Work

In this work, a convex optimization model was presented to learn a health index of a battery. Such a health index can accurately capture the degradation trajectory of a battery as well as help improve SOH prediction accuracy. Moreover, we developed an attention-based LSTM algorithm for predicting the SOH of a battery, where an attention matrix, referring to the significance level of a time series, was adopted so that the predictive model can utilize the most significant portion of a time series while predicting. A battery dataset, including current, voltage, and temperature, collected from four batteries was utilized to evaluate the performance of the presented algorithm. The numerical results have illustrated that the learned health indices can precisely capture the capacity fade and regeneration phenomenons of the four batteries. The results have also shown that utilizing the learned health index can improve the SOH prediction performance as the average RMSE with utilizing the health index is 0.0157 in comparison with the average RMSE without utilizing the health index is 0.0164 when over 20 cycles have been observed. We can also conclude that using the attention mechanism can also achieve a better SOH estimation performance. For example, with respect to No. 7, the RMSE of the presented algorithm is 0.0140, however, the RMSE of HI-LSTM is 0.0447. In addition, the presented algorithm outperforms other algorithms used in the literature.

The field of battery research is constantly evolving, and future efforts are expected to focus on predicting the SOH of batteries across a wide range of different battery types. Currently, much of the research in this area has been focused on lithium-ion batteries, which are widely used in a variety of applications, including electric vehicles, smartphones, and laptops. However, there are many other types of batteries, including lead-acid, nickel-cadmium, and zinc-carbon batteries, which also require accurate and reliable SOH prediction methods. Research in this area is likely to involve developing new algorithms and machine learning models that can accurately predict the remaining capacity and useful lifetime of these batteries, as well as identifying the factors that contribute to degradation and failure. In addition to improving SOH prediction in different battery types, future research is also expected to facilitate the detection of small devices. As technology becomes increasingly miniaturized, there is a growing need for small, reliable batteries that can power these devices for extended periods of time. Research in this area is likely to focus on developing new battery chemistries that can deliver high energy densities in small form factors, as well as improving the accuracy and sensitivity of diagnostic tools for detecting small battery defects and failures. Overall, the future of battery research is likely to be focused on developing more accurate and reliable methods for predicting SOH across a wide range of battery types, as well as improving the performance and reliability of small batteries for use in miniature devices.

## Figures and Tables

**Table 1:** Hyperparameters and network architecture utilized in this case study for SOH predictions.

Sequence of Layers	Description	Output Dimensionality
1	Input layer	30×165×9
2	LSTM layer	30×165×50
3	Attention layer	30×5×50
4	Flatten layer	30×250
5	Output layer	30×1

**Table 2:** Symbols and descriptions of the presented algorithm and comparable algorithms.

Method Symbol	Method Description
HI-ALSTM	Health index informed attention-based LSTM model (Proposed methodology)
HI-LSTM	Health index-informed LSTM model without using the attention mechanism
ALSTM	Attention-based LSTM predictive model without using the health index
LSTM	traditional LSTM predictive model

**Table 3:** The MAE of the proposed algorithm and algorithms listed in Table 2 for all battery units.

	Battery No. 5	Battery No. 6	Battery No. 7	Battery No. 18	Average
HI-ALSTM	0.0149	0.0110	0.0068	0.0083	0.0103
HI-LSTM	0.0380	0.0551	0.0466	0.0222	0.0405
ALSTM	0.0066	0.0170	0.0133	0.0114	0.0121
LSTM	0.0362	0.0213	0.0372	0.0264	0.0303

**Table 4:** The MAPE of the proposed algorithm and algorithms listed in Table 2 for all battery units.

	Battery No. 5	Battery No. 6	Battery No. 7	Battery No. 18	Average
HI-ALSTM	196.30%	151.03%	84.17%	108.29%	134.95%
HI-LSTM	512.55%	793.49%	611.81%	292.07%	552.48%
ALSTM	85.89%	247.20%	165.64%	150.38%	162.28%
LSTM	487.97%	290.07%	485.74%	352.43%	404.05%

**Table 5:** The RMSE of the proposed algorithm, algorithms listed in Table 2, and other algorithms reported in the literature for all battery units.

	Battery No. 5	Battery No. 6	Battery No. 7	Battery No. 18	Average
HI-ALSTM	0.0165	0.0153	0.0089	0.0139	0.0136
HI-LSTM	0.0399	0.0612	0.0542	0.0274	0.0457
ALSTM	0.0085	0.0220	0.0145	0.0151	0.0150
LSTM	0.0375	0.0249	0.0427	0.0288	0.0335
LR-GPR [44]	0.0168	0.0292	-	0.0169	0.0210
GBDT [45]	0.0192	0.0281	0.0157	-	0.0210

## Data Availability

The dataset used in this study are available at https://www.nasa.gov/content/prognostics-center-of-excellence-data-set-repository, accessed on 01 December 2022.

## Abbreviations

The following abbreviations are used in this manuscript:

HI	Health Index
SOH	State of Health
CC	Constant Current
CV	Constant Voltage
PCoE	Prognostics Center of Excellence
RSME	Root Mean Squared Error
LSTM	Long Short-term Memory
FC	Fully Connected
